# Collagen Fingerprinting and the Earliest Marine Mammal Hunting in North America

**DOI:** 10.1038/s41598-018-28224-0

**Published:** 2018-07-03

**Authors:** Courtney A. Hofman, Torben C. Rick, Jon M. Erlandson, Leslie Reeder-Myers, Andreanna J. Welch, Michael Buckley

**Affiliations:** 1 Department of Anthropology, University of Oklahoma, 455 W. Lindsey St., Norman, OK 73019 USA; 2 Center for Conservation Genomics, Smithsonian Conservation Biology Institute, National Zoological Park, MRC 5513, Washington, DC 20013-7012 USA; 3 Department of Anthropology, National Museum of Natural History, MRC 112, Smithsonian Institution, Washington, DC 20013-7012 USA; 4 Museum of Natural and Cultural History, University of Oregon, Eugene, OR 97403 USA; 5 Department of Anthropology, Temple University, Philadelphia, PA 19122 USA; 6 Department of Biosciences, Durham University, South Road, Durham, DH1 3LE UK; 7 School and Earth and Environmental Sciences, Manchester Institute of Biotechnology, 131 Princess Street, University of Manchester, Manchester, M1 7DN UK

## Abstract

The submersion of Late Pleistocene shorelines and poor organic preservation at many early archaeological sites obscure the earliest effects of humans on coastal resources in the Americas. We used collagen fingerprinting to identify bone fragments from middens at four California Channel Island sites that are among the oldest coastal sites in the Americas (~12,500-8,500 cal BP). We document Paleocoastal human predation of at least three marine mammal families/species, including northern elephant seals (*Mirounga angustirostris*), eared seals (Otariidae), and sea otters (*Enhydra lutris*). Otariids and elephant seals are abundant today along the Pacific Coast of North America, but elephant seals are rare in late Holocene (<1500 cal BP) archaeological sites. Our data support the hypotheses that: (1) marine mammals helped fuel the peopling of the Americas; (2) humans affected marine mammal biogeography millennia before the devastation caused by the historic fur and oil trade; and (3) the current abundance and distribution of recovering pinniped populations on the California Channel Islands may mirror a pre-human baseline.

## Introduction

Recent archaeological, genomic, and paleoecological research identifies the Pacific Coast as one of the gateways for the peopling of the Americas^1–8^. California’s Channel Islands figure prominently in this research with ~13,000-11,000 year old sites that contain human remains, sophisticated hunting tools, and diverse faunal assemblages^3,9,10^. These island Paleocoastal sites have helped reframe debates about the nature of human use of coastal ecosystems along the Pacific Coast and around the world^11,12^.

An important research area in coastal archaeology focuses on the interactions between people and marine mammals. This includes the role of marine mammals as a food source, the implications of marine mammal hunting for understanding the impact of humans on newly colonized ecosystems, and how these data can help inform marine mammal conservation efforts. Some scholars have speculated that pinnipeds (seals and sea lions), with their large size and use of onshore haulouts, were attractive and vulnerable to early coastal hunters^13,14^, but the submergence of early coastal sites by rising postglacial seas limits knowledge of early maritime hunting practices and ancient pinniped populations^3^. In early Pacific Coast archaeological sites, seal and sea lion bones are generally rare, highly fragmented, and mostly unidentifiable to species. For instance, at a Late Pleistocene site on Isla Cedros, Baja California, a single Guadalupe fur seal (*Arctocephalus townsendi*) bone dates to ~11,070-10,680 cal BP, but fish appear to dominate the assemblage^15^. A larger assemblage from the ~9400 year old Kilgi Gwaii site on Haida Gwaii, British Columbia, includes harbor seal (*Phoca vitulina*), sea otter (*Enhydra lutris*), and Stellar sea lion (*Eumetopias jubatus*) bones but, similar to Baja California, fish and birds were significantly more abundant^4^. In South America, Dillehay *et al*. (2017) recently identified 39 marine mammal bones from Late Pleistocene localities including a direct date of roughly 13,300 cal BP, a few centuries older than the oldest specimens in North America^16^. All these bones are from *Otaria* sp. or *Otaria flavescens* that may have been clubbed while hauled out onshore or scavenged from carcasses washed ashore.

The dearth of identified bones from Paleocoastal sites limits our understanding of the structure of Late Pleistocene and early Holocene marine mammal communities and the potential effects of human lifeways on marine mammal populations^17–22^. Known Late Pleistocene archaeological sites are often several kilometers from the submerged ancient shorelines, and people would likely have butchered carcasses near the shore rather than hauling them to interior camps^3^. Due to these processing and transport issues, as well as fragmentation of faunal remains at some sites, potential marine mammal bones are often undiagnostic fragments that could be from pinnipeds, sea otters, or cetaceans. Better taxonomic identification of marine mammals in early archaeological sites would provide a more precise picture of the species available for human consumption along the Pacific Coast when people first arrived and the potential impact of humans on pinniped populations in the Late Pleistocene.

Today, many marine mammal populations have recovered from near extinction during the historical fur and oil trade and their recovery is a major conservation success. However, the long-term context for that recovery is important for understanding the implications of recent shifts in species composition from the late Holocene to the present^13,17–23^. We seek to address this gap by using collagen fingerprinting to identify fragmentary marine mammal remains from Paleocoastal sites on Alta California’s Channel Islands to understand the nature of Paleocoastal marine mammal exploitation and how these data compare to late Holocene and modern marine mammal distributions and abundance.

## Results

**Figure 1:**
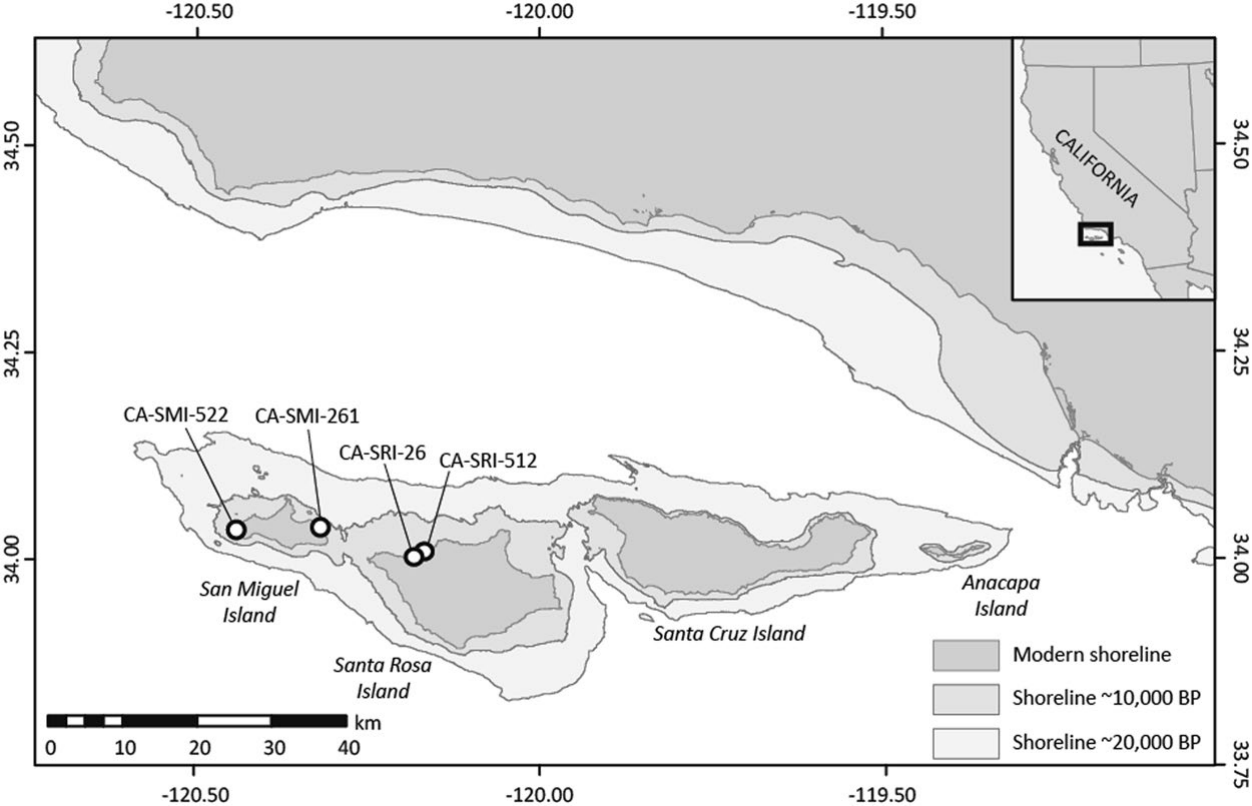
Paleocoastal archaeological sites on California’s northern Channel Islands. Four archaeological sites span two different islands today but at the Last Glacial Maximum (LGM) and as recently as 9,000 years ago, the northern Channel Islands coalesced into a single landmass called Santarosae. (Paleo-shorelines from Reeder-Myers *et al*. 2015^39^).

**Figure 2:**
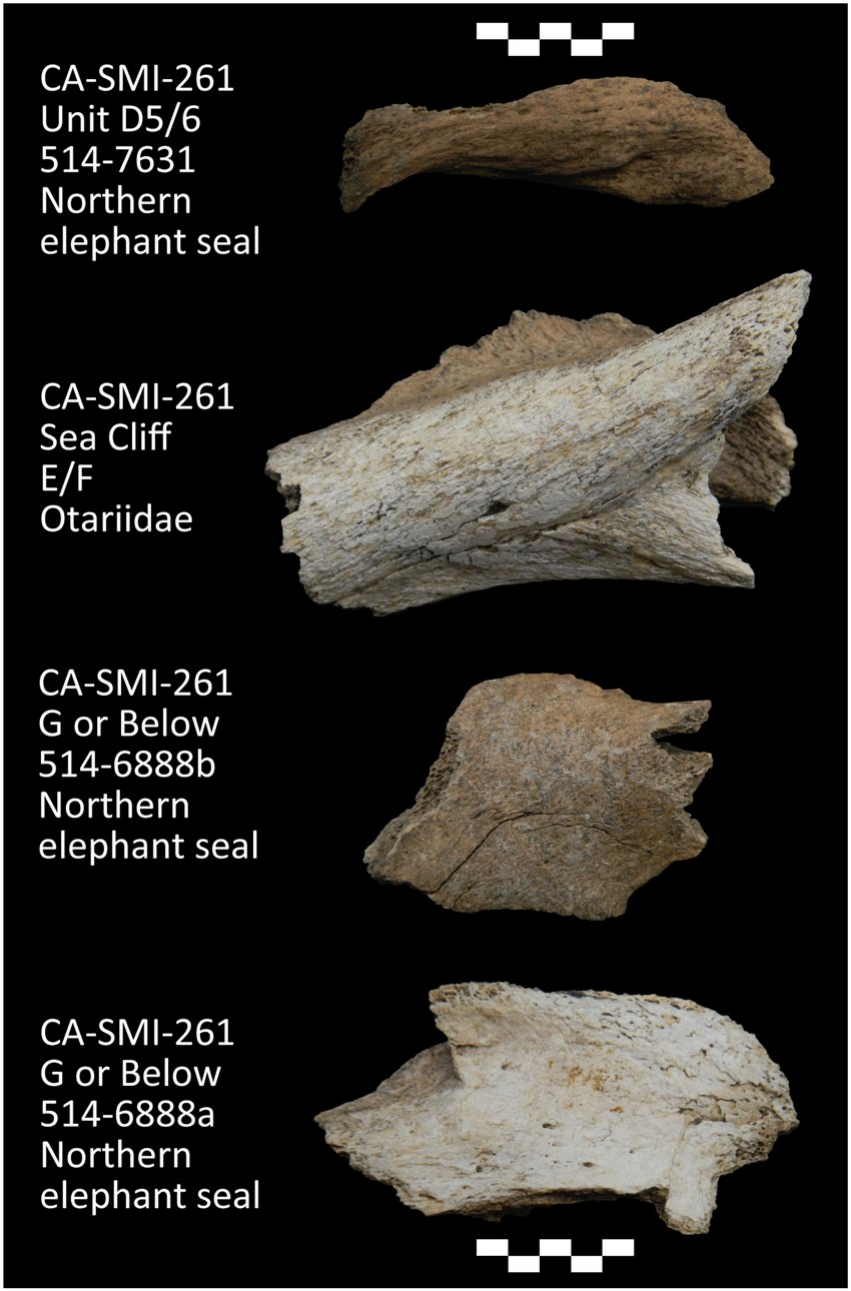
Sample of the fragmented marine mammal bones analyzed and identified in this study from archaeological contexts.

**Figure 3:**
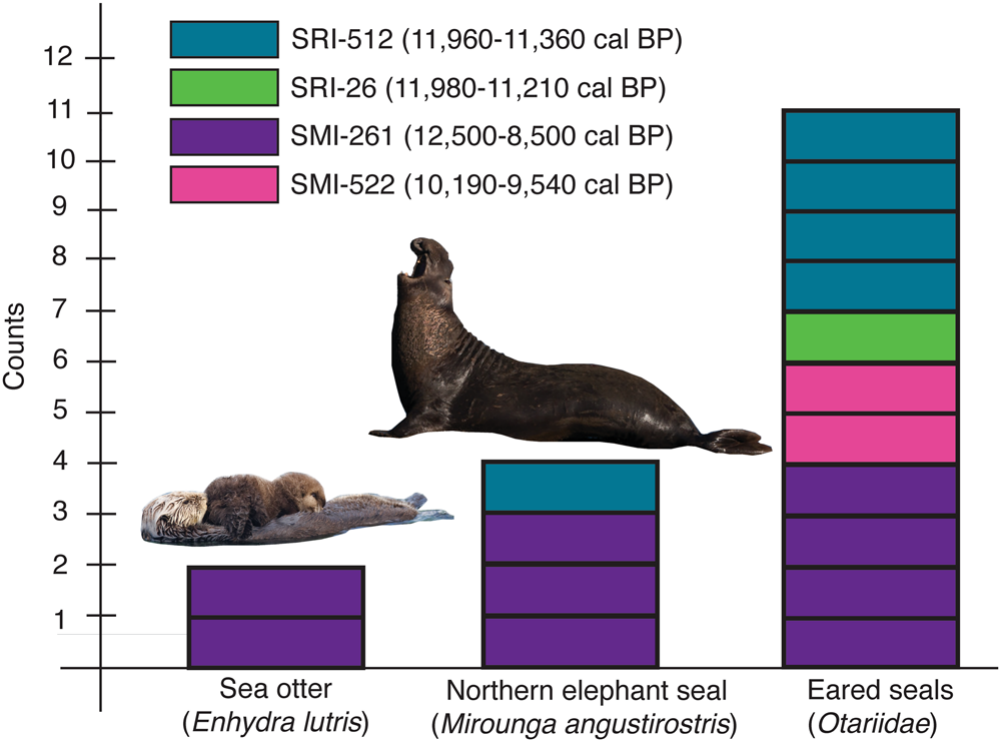
Species identification of fragmented bone samples from Paleocoastal archaeological sites. Collagen fingerprinting identified six samples to species and eleven samples to family. Northern elephant seals are present in two different archaeological sites but are rare in the archaeological record for most of the Holocene.

We performed collagen fingerprinting taxonomic identification of 20 unidentified marine mammal bone fragments from four Paleocoastal sites on the Channel Islands dated between ~12,500-8500 cal BP. When occupied, the sites were located within 10 km of the northern coast of Santarosae Island (Fig. 1). Our sample size is relatively small, but bone fragments were selected from stratified and well-dated excavation contexts (discrete strata, test units, etc.) to minimize the potential of sampling the same individual (Fig. 2, Table 1). For taxonomic identification, collagen fingerprints of bone fragments were compared to known collagen fingerprints from modern specimens (Supplementary Information Figs S1–3). Seventeen of the 20 bone samples yielded identifiable collagen fingerprints, six specimens were identified to species, eleven to the family Otariidae (eared seals), and three produced ambiguous fingerprints or poor collagen (Fig. 3). The difficulty in differentiating collagen fingerprints in ancient samples and modern comparative specimens of Otariidae is unsurprising given the recent radiation of the family^24^. Marine mammal biogeography narrows the otariid samples to four likely species with prehistoric or extant distributions in this region: Guadalupe fur seal, northern fur seal (*Callorhinus ursinus*), California sea lion (*Zalophus californianus*), and Steller sea lion.

**Table 1:** Sample context, species identification and age.

Site No. (CA-)	Provenience	Stratum/Level	Catalog Number	ZooMS ID	Date (cal BP)^1^
SMI-261	Column E6	E1	514–380b	Otariidae	~8900-8500
SMI-261	Unit D5	E2	514–5710	Sea otter	~8900-8500
SMI-261	D5/6	E2b	514–7631	Elephant seal	~8900-8500
SMI-261	Unit 98- E6	E3	514–7787	Ambiguous	~9200-8900
SMI-261	Column E6	E3	514–7700b	Otariidae	~9200-8900
SMI-261	Profile E6	E	514–7919	Otariidae	~9200-8500
SMI-261	Sea cliff	E/F	6908	Otariidae	10,130-8500
SMI-261	Sea cliff	E/F		Ambiguous	10,130-8500
SMI-261	Unit E6	F1	514–6809b	Sea otter	9630-9150
SMI-261	Sea cliff	G or below	514–6888b	Elephant seal	12,500-11,220
SMI-261	Sea cliff	G or below	514–6888a	Elephant seal	12,500-11,220
SMI-522	Sea cliff	Paleocoastal	522–78b	Otariidae	10,190-9540
SMI-522	25	2	522–247b	Otariidae	10,190-9540
SRI-26	Unit 2	5		Otariidae	11,980-11,210
SRI-26	Gully wall	A4		Poor collagen	11,980-11,210
SRI-512	Unit 3	profile	512–369b	Elephant seal	11,960-11,360
SRI-512	Unit 4	5		Otariidae	11,960-11,360
SRI-512	Unit 4	4	512–419Ab	Otariidae	11,960-11,360
SRI-512	Unit 7	2	Feature 1	Otariidae	11,960-11,360
SRI-512	Unit 6	39-43 cm		Otariidae	11,960-11,360

^1^Dates are calibrated AMS radiocarbon dates from associated material in the level.

At CA-SMI-261 (Daisy Cave), three elephant seal (*Mirounga angustirostris*) bones were identified: two from a terminal Pleistocene stratum (12,500-11,220 cal BP) that may be from a single individual and one dated to ~8900-8500 cal BP, along with two sea otter bones dated to the early Holocene and four otariids. One of these elephant seal fragments (514-6888b) exhibited possible cutmarks indicative of human processing. CA-SMI-522 yielded two otariids dated to ~10,190–9540 cal BP. Six bone fragments were identified from the Santa Rosa Island sites, CA-SRI-26 and CA-SRI-512, both dated between ~12,000-11,210 cal BP. An elephant seal bone was identified at CA-SRI-512, along with four otariid bones. A single bone from CA-SRI-26 yielded enough collagen to be identified as an otariid.

## Discussion

The marine mammals identified in our Paleocoastal assemblages are among the earliest evidence for marine mammal exploitation in North America. Questions remain about whether the pinnipeds and sea otters found at these Paleocoastal sites were hunted or scavenged. To date, at least 10 Channel Island sites ≥10,000 years have produced marine faunal remains associated with finely-crafted projectile points (including the four sites reported here), suggesting active hunting of marine resources during this period^2,3^. One elephant seal specimen from CA-SMI-261 has possible cutmarks, suggesting processing of the marine mammal remains (Fig. 2). The fragmentary nature of bones recovered in these sites and the small proportion of marine mammal bones relative to other taxa, however, make it difficult to determine if these animals were taken through direct hunting or scavenging. Given the presence of boats and other sophisticated Paleocoastal technologies, as well as the variety of marine mammals present in these sites, people clearly had the capability to hunt pinnipeds and sea otters by the Late Pleistocene. Many of the marine mammals from these sites may have been hunted, but scavenging may also have occurred.

Archaeologists have built a relatively detailed picture of later Holocene (≤1500 cal BP) ancient marine mammal hunting for the California Channel Islands and Pacific Coast^13,17–20,22,23,25–28^. The remains of Guadalupe fur seals, California sea lions, and sea otters dominate these later assemblages from the southern California Bight^18,19,22,23,25,27^. Although elephant seals have relatively robust bones that should preserve well, they are rare in these late archaeological assemblages from the California Coast, contributing just 0.6% of identified pinniped bones from dozens of sites and thousands of bones^19,23^. Otariid remains were present in all four Paleocoastal sites and were more common in our sample than elephant seals or sea otters, a pattern that could be related to higher encounter rates with otariids, as well as issues of the greater ease of interior transport of otariid remains. The occurrence of four elephant seal bones in two of the four Paleocoastal sites is surprising, however, especially given challenges of transporting large marine mammals, even processed animals, to the inshore localities where the bones were found.

The presence of elephant seals within this sample from the Pleistocene/Holocene transition indicates that they were an accessible resource for Paleocoastal peoples. Northern elephant seals suffered a significant population bottleneck during historic commercial hunting, but have since recovered and are common on the Channel Islands^29–31^. Elephant seals are rare in late Holocene sites, suggesting that modern pinniped communities have significantly reorganized since historic decline^19,22^. Northern elephant seals prefer sandy beaches for thermal regulation, limiting them to prime habitat also occupied by early humans^19^. Elephant seal pups—left virtually helpless on beaches while mothers feed at sea—would have been especially vulnerable to hunters. While the sample size is small, our data raise the possibility that human activities may have influenced pinniped community structure on the Channel Islands as early as the terminal Pleistocene or early Holocene, reducing the number of elephant seals, displacing them to inaccessible offshore islands and remote pocket beaches, or both^19,23^.

During the 19^th^ century, numerous Pacific Coast marine mammals were hunted nearly to extinction by the commercial fur and oil trade. Molecular data document an extreme loss of biodiversity in elephant seals, fur seals, and sea otters during this period^30–34^. In spite of near extirpation, more than 100,000 individuals from six pinniped species spend time on land or breed on California’s Channel Islands each year^29^. Given their historic population collapse and rapid recovery, questions remain about the prehistoric biogeography of these marine species. Some have recovered dramatically under legal protection, with more than 179,000 elephant seals in California today and larger numbers of California sea lions^29^. The Guadalupe fur seal, in contrast, remains rare north of Mexico^18,23^. Our collagen fingerprinting data suggest that these species may be returning to a structure similar to that of the terminal Pleistocene, prior to millennia of intensive hunting by Native Americans and later commercial hunting by Europeans and Euro-Americans.

More data are required to test this hypothesis but collagen fingerprinting provides valuable insights into the nature of Paleocoastal marine hunting on the Channel Islands and contributes to our understanding of pinniped biogeography across the Pleistocene-Holocene boundary. Applied globally to other time periods and species, collagen fingerprinting can increase understanding of human coastal adaptations and help guide the conservation and management of a suite of threatened marine mammals and other wildlife around the world.

## Materials and Methods

### Context and Chronology

All samples we analyzed were obtained during systematic archaeological excavations at four well-dated archaeological sites that contain no evidence of stratigraphic mixing and have been well reported in the archaeological literature (Supplementary Information, Table S2). Sample date ranges were obtained from 36 calibrated ^14^C dates associated with Paleocoastal components that produced marine mammal remains; 10 from CA-SMI-522, 17 from Paleocoastal components at CA-SMI-261, 6 from CA-SRI-512, and 3 from CA-SRI-26^3,35–37^. Each site produced vertebrate and shellfish remains, with marine mammal remains generally occurring as small, unidentifiable fragments. At CA-SRI-512, 5,534 fish, bird, and mammal bone fragments were recovered, including 91 pinniped bone fragments, but only one of these was identified beyond family level, a possible harbor seal bone^3^. Similarly, at CA-SMI-522, just two marine mammal bones were identified but none to species^35^. Final vertebrate data from CA-SMI-261 have yet to be reported but only a single sea otter bone had been identified among a relatively small assemblage of pinniped bone fragments. At CA-SRI-26 a small assemblage (<100) of highly fragmented marine mammal bones includes no diagnostic elements. We chose 20 samples for our analysis that were relatively well-preserved and from secure stratigraphic contexts that provided temporal and spatial coverage.

### Collagen Fingerprinting

Fragmented bones were sent to the University of Manchester for collagen fingerprinting. Collagen was extracted following previously published methods^38^, with ~50 mg bone powder demineralized with 1 mL 0.6 M hydrochloric acid overnight and, following centrifugation at 13,000 rpm, the acid-soluble fraction ultrafiltered into 100 µL 50 mM ammonium bicarbonate (ABC). This solution was digested with 0.4 µg sequencing grade trypsin at 37 °C for 18 hours. The resultant peptide solution was purified using C18 ziptips and dried by centrifugal evaporation. Samples were resuspended with 10 µL 0.1% trifluoroacetic acid and 1 µL co-crystalized onto a stainless steel Matrix Assisted Laser Desorption Ionisation (MALDI) target plate with a 1 µL a-cyano hydroxycinnamic acid matrix. MALDI analysis utilized a Bruker Ultraflex II mass spectrometer using 2,000 laser acquisitions.

Reference spectra for *Mirounga leonina*, *Mirounga angustirostris*, *Phoca vitulina*, *Arctocephalus australis*, *Arctocephalus gazella*, *Eumotopias jubatus*, *Otaria flavescens*, *Enhydra lutris*, *Callorhinus ursinus*, *Zalophus californianus*, *Odobenus rosmarus*, and *Arctocephalus townsendi* (Supplementary Information Figs 1–3) were compared with those of previous publications to establish potential new biomarkers (Table S1), which were then compared with the archaeological spectra (Supplementary Information Figs 4–8). Species level designations were determined by marine mammal biogeography. Ambiguous fingerprints were generated in two samples. Due to the presence of one of the trypsin peaks at m/z 2211, one sample was determined to have poor collagen preservation.

### Data availability

The datasets supporting this article have been uploaded as part of the supplementary material.

## Electronic supplementary material

Supplementary Information
