# Supplementary material for: Collagen Fingerprinting and the Earliest Marine Mammal Hunting in North America
Source: Sci Rep. 2018 Jul 3;8:10014. doi: 10.1038/s41598-018-28224-0 (PMC6030183; doi:10.1038/s41598-018-28224-0)
Supplement: Supplementary file 1 — Supplementary Information [file 41598_2018_28224_MOESM1_ESM.pdf]

Supplementary Information: Collagen Fingerprinting and the Earliest Marine Mammal Hunting in North America

Authors: Courtney A. Hofman[1,2*], Torben C. Rick[3], Jon M. Erlandson[4], Leslie Reeder-Myers[5] Andreanna J. Welch[6,2] and Michael Buckley[7*]

Affiliations:
[1]Department of Anthropology, University of Oklahoma, 455 W. Lindsey St., Norman, OK 73019, courtney.hofman@ou.edu

[2]Center for Conservation Genomics, Smithsonian Conservation Biology Institute, National Zoological Park, MRC 5513, Washington, DC, 20013-7012

[3]Department of Anthropology, National Museum of Natural History, MRC 112, Smithsonian Institution, Washington D.C. 20013-7012, rickt@si.edu

[4]Museum of Natural and Cultural History, University of Oregon, Eugene, OR, 97041, jerland@uoregon.edu

[5]Department of Anthropology, Temple University, Philadelphia, PA, 19122, leslie.reeder-myers@temple.edu

[6]Department of Biosciences, Durham University, South Road, Durham, DH1 3LE, UK, a.j.welch@durham.ac.uk

[7]School and Earth and Environmental Sciences, Manchester Institute of Biotechnology, 131 Princess Street, University of Manchester, Manchester, M1 7DN, UK, m.buckley@manchester.ac.uk

*Correspondence to: courtney.hofman@ou.edu and m.buckley@manchester.ac.uk

Photo credits:
Figure 3 utilized two images from the public domain :
"Mike" Michael L. Baird, flickr.bairdphotos.com
https://www.flickr.com/photos/mikebaird/5379599365/in/photostream/ (Own work, CC-BY 2.0).

"Mike" Michael L. Baird, flickr.bairdphotos.com
https://commons.wikimedia.org/wiki/File:Sea_otter_nursing_young.jpg
https://www.flickr.com/photos/72825507@N00/2979351573/ (Own work, CC-BY 2.0).

Chronology:
The marine mammal samples in this study are from four well dated Paleocoastal sites, with stratified and secure Early Holocene or Terminal Pleistocene deposits and radiocarbon chronologies. Table S1 presents all Paleocoastal ^{14}C dates from each of the cultural components at the four archaeological sites from which our samples were selected. Additional details of the stratigraphic context of the samples and site chronologies are available in Erlandson et al. (1996, 2011) and Watts (2013). All ^{14}C dates were calibrated using CALIB 7.1, with the Marine13 dataset for all shell samples and the INTCAL13 dataset for terrestrial samples (Reimer et al. 2013). A ΔR of 261 ± 21 (Jazwa et al. 2012) was applied to all marine samples.

References
Erlandson, J. M., D. J. Kennett, B. L. Ingram, D. A. Guthrie, D. P. Morris, M. A. Tveskov, G. J. West, and P. L. Walker. 1996. An archaeological and paleontological chronology for Daisy Cave (CA-SMI-261), San Miguel Island, California. *Radiocarbon* 38:355–373.
Erlandson, J. M., T. C. Rick, T. J. Braje, M. Casperson, B. Culleton, B. Fulfrost, T. Garcia, D. A. Guthrie, N. Jew, D. J. Kennett, M. L. Moss, L. A. Reeder, C. E. Skinner, J. Watts, and L. Willis. 2011. Paleoindian seafaring, maritime technologies, and coastal foraging on California's Channel Islands. *Science* 331:1181–1185.
Jazwa, C., D. Kennett, and D. Hanson. 2012. Late Holocene subsistence change and marine productivity on western Santa Rosa Island, Alta California. *California Archaeology* 4:69–98.
Reimer, P. J., E. Bard, A. Bayliss, J. W. Beck, P. G. Blackwell, C. Bronk Ramsey, C. E. Buck, H. Cheng, R. L. Edwards, M. Friedrich, P. M. Grootes, T. P. Guilderson, H. Haflidason, I. Hajdas, C. Hatte, T. H. Heaton, D. L. Hoffman, A. Hogg, K. A. Hughen, K. F. Kaiser, B. Kromer, S. W. Manning, M. Niu, R. W. Reimer, D. A. Richards, E. M. Scott, J. R. Southon, R. A. Staff, C. S. M. Turney, and J. van der Plicht. 2013. IntCal13 and Marine13 radiocarbon age calibration curves 0-50,000 years cal BP. *Radiocarbon* 55:1869–1887.
Watts, J. L. 2013. *The Culture if Santarosae: Subsistence Strategies and Landscape Use in the Northern Channel Islands from the Initial Occupation*. PhD Dissertation, Oxford University.

Table S1. Paleocoastal radiocarbon data from CA-SMI-261, CA-SMI-522, CA-SRI-26 and CA-SRI-512.

**Provenience**	**Lab Number**	**Material**	**$^{13}C/^{12}C$ Adjusted**	**Age Range (cal BP, 1σ)**
**CA-SMI-261**				
Col. E6, Str. E1	CAMS-8866	Charred twig	7810 ± 60	8680-8480

Col. E6, Str. E1	CAMS-14379	CA mussel	8380 ± 60	8690-8500
Col. E6, Str. E1	CAMS-14380	CA mussel	8400 ± 60	8710-8520
Col. E6, Str. E1	CAMS-14360	CA mussel	8440 ± 80	8830-8550
Col. E6, Str. E1	Beta-15621	Black abalone	8460 ± 100	8870-8570
Col. E6, Str. E1	CAMS-14361	CA mussel	8500 ± 80	8900-8630
Col. E6, Str. E4	Beta-15622	Black abalone	8730 ± 120	9280-8950
Col. E6, Str. E4	CAMS-8865	Charred twig	8040 ± 60	9020-8780
Col. E6, Str. F1	Beta-15623	CA mussel	8900 ± 120	9430-9150
Col. E6, Str. F1	CAMS-8867	Charred twig	8600 ± 60	9630-9520
Col. E6, Str. F3	Beta-49948	CA mussel	9360 ± 90	10,000-9670
Col. E6, Str. F3	CAMS-8863	Charred twig	8810 ± 80	10,130-9690
Col. E6, Str. F/G	CAMS-33368	Charcoal	8970 ± 60	10,230-9950
Col. E6, Str. F/G	CAMS-33375	Marine shell	9620 ± 70	10,280-10120
Col. E6, Str. G	Beta-52360	Black turban	10,600 ± 70	11,540-11,220
Col. E6, Str. G	Beta-14660	Red abalone	10,700 ± 90	11,750-11,350
Col. E6, Str. G	CAMS-9094	Wood charcoal	10,390 ± 130	12,520-12,050
**CA-SMI-522**				
1S-III-A	OS-68509	Marine shell	9230 ± 40	9670-9540
1N-7	OS-68511	Marine shell	9240 ± 40	9690-9550
1S-IV	OS-68510	Marine shell	9260 ± 55	9740-9550
IS-A	OS-68512	Marine shell	9300 ± 45	9830-9630
1S Wall-2	OS-68508	Marine shell	9330 ± 45	9880-9680
1S-B, (Notes I)	OS-69026	Charred twig	8780 ± 50	9900-9700
Sea cliff: base of midden	OS-37737	Marine shell	9390 ± 30	9990-9770
Above base	OS-27943	*Mytilus californianus*	9450 ± 70	10,120-9880
Sea cliff: base of midden	OS-37963	Charred twig	8870 ± 40	10,150-9910
1S-III-B	OS-69027	Charred twig	8940 ± 50	10,190-9940
**CA-SRI-26**				
Gully, A4 soil, ~2 m below surface	UCI-80937	*Mytilus californianus*	10,545 ± 30	11,310-11,210
A4 Paleosol	DAMS-8725	*Haliotis rufescens*	10,700 ± 37	11,690-11,390
A4 Paleosol	OS-96885	Goose bone (uf)	10,150 ± 70	11,980-11,640
**CA-SRI-512**				
A5 Paleosol	UCIAMS-59871	Goose bone (XAD extract)	10,000 ± 30	11,600-11,360

A5 Paleosol	UCIAMS-59872	Goose bone (XAD extract)	10,045 ± 40	11,700-11,400
Below A5	Beta-261353	*Ceanothus* charcoal	10,090 ± 50	11,810-11,410
A5 Paleosol	UCIAMS-60751	Charred twig	10,160 ± 30	11,950-11,760
A5 Paleosol	OS-68030	Goose bone (ultrafiltration)	10,150 ± 40	11,960-11,720
Below A5	OS-75147	*Ceanothus* charcoal	10,200 ± 45	12,000-11,811

Table S2. Table of ZooMS markers useful for identifying Marine Mammals.

**Common Name**	**Species name**	**A**	**B**	**C**	**D**	**P**	**ES***	**F**	**G**
Southern Elephant seal	*Mirounga leonina*	1205/1221	1453	1566	2171	2215	2663	2853	2957
Northern Elephant seal	*Mirounga angustirostris*	1205/1221	1453	1566	2171	2215	2663	2853	2957
Harbor Seal	*Phoca vitulina*	1205/1221	1453	1566	2171	2215	2705	2853	2957
South American Fur Seal	*Arctocephalus australis*	1205/1221	1453	1566	2121	2215	2705	2853	2957
Antarctic Fur Seal	*Arctocephalus gazella*	1205/1221	1453	1566	2121	2215	2705	2853	2957
Stellar's Sea Lion	*Eumotopias jubatus*	1205/1221	1453	1566	2121	2215	2705	2853	2957
South American Sea Lion	*Otaria flavescens*	1205/1221	1453	1566	2121	2215	2705	2853	2957
Northern Fur Seal	*Callorhinus ursinus*	1205/1221	1453	1566	2121	2215	2705	2853	2957
California Sea Lion	*Zalophus californianus*	1205/1221	1453	1566	2121	2215	2705	2853	2957
Walrus	*Odobenus rosmarus*	1205/1221	1453	1566	2121	2246	2705	2853	3003
Guadalupe Fur Seal	*Arctocephalus townsendi*	1205/1221	1453	1566	2121	2246	2705	2853	2957
Sea Otter	*Enhydra lutris*	2119/1235	1453	1566	2147	2215	2705	2853	2973

*Marker described here as useful for identifying Elephant Seals

Figures S1-S3. Collagen Fingerprint Reference Spectra for Marine Mammal Identification
Figure S4-S8. Collagen Fingerprint Spectra for Samples in this Study

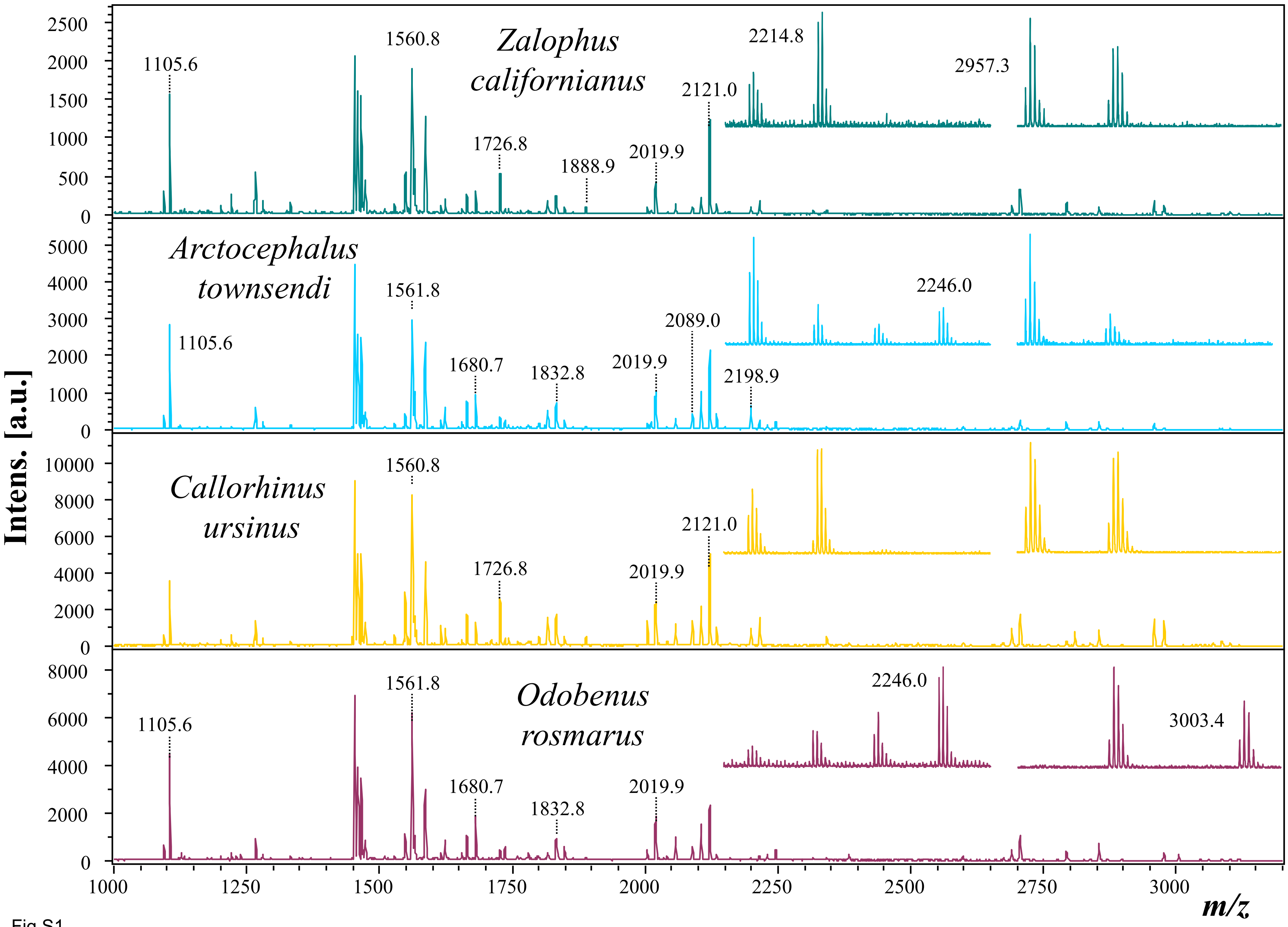
Zalophus californianus
Arctocephalus townsendi
Callorhinus ursinus
Odobenus rosmarus
1105.6
1560.8
1726.8
1888.9
2019.9
2121.0
2214.8
2957.3
1561.8
1680.7
1832.8
2089.0
2198.9
2246.0
3003.4
Intens. [a.u.]
m/z

Fig S1

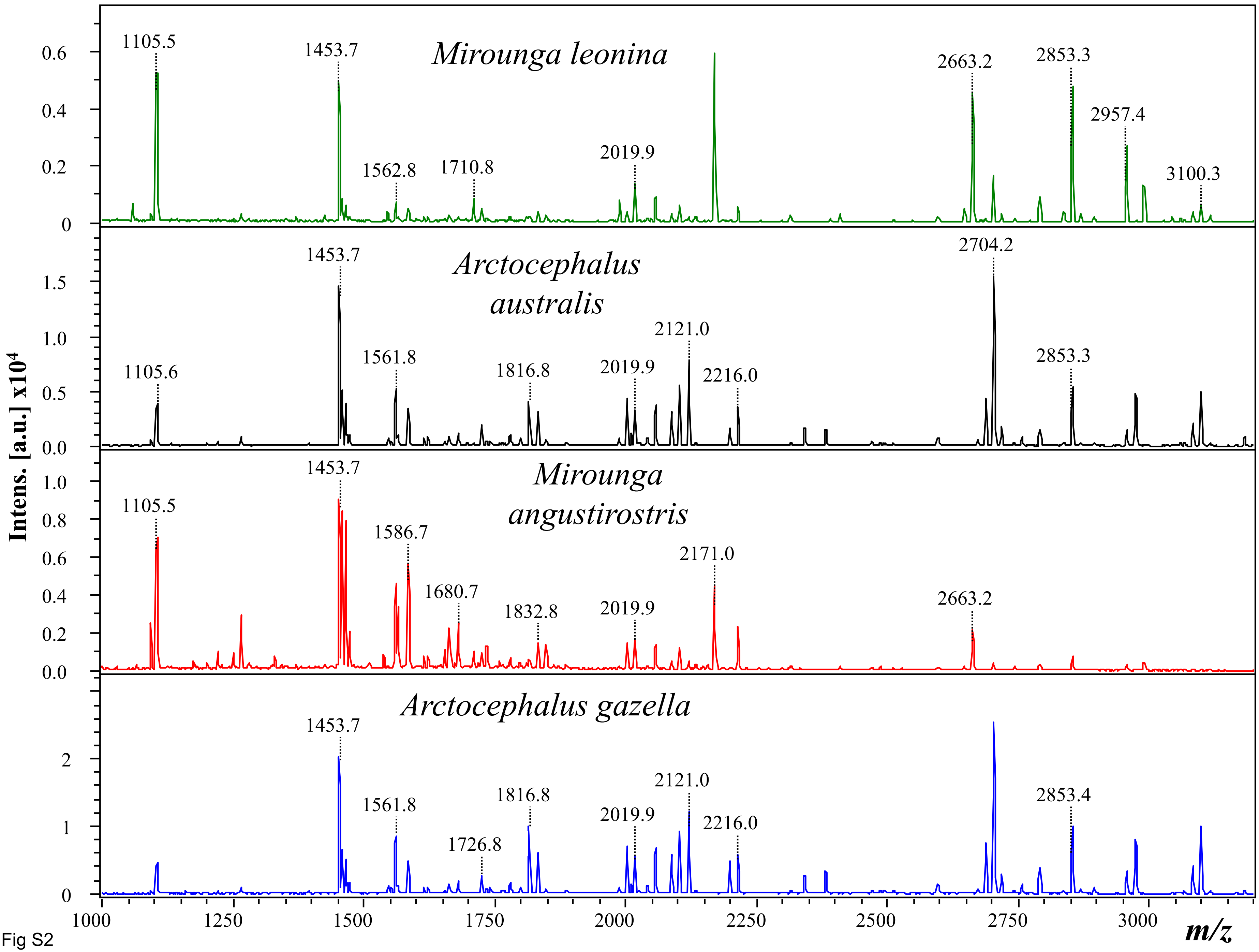
Mirounga leonina
1105.5
1453.7
1562.8
1710.8
2019.9
2663.2
2853.3
2957.4
3100.3
Arctocephalus australis
1105.6
1453.7
1561.8
1816.8
2019.9
2121.0
2216.0
2704.2
2853.3
Mirounga angustirostris
1105.5
1453.7
1586.7
1680.7
1832.8
2019.9
2171.0
2663.2
Arctocephalus gazella
1453.7
1561.8
1726.8
1816.8
2019.9
2121.0
2216.0
2853.4
Intens. [a.u.] x10^4
m/z

Fig S2

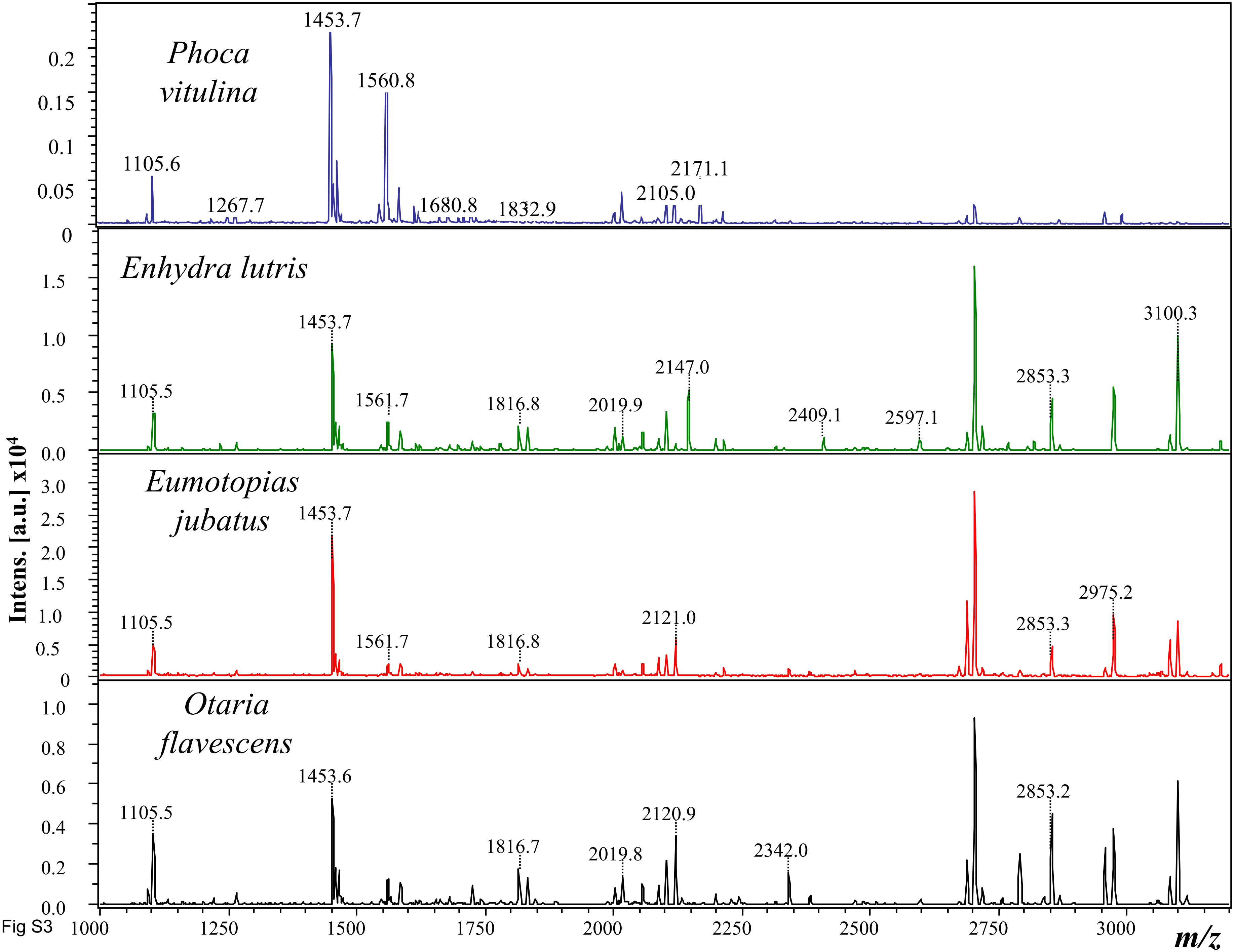
Phoca vitulina
1453.7
1560.8
1105.6
1267.7
1680.8
1832.9
2105.0
2171.1
Enhydra lutris
1453.7
3100.3
2147.0
2853.3
1105.5
1561.7
1816.8
2019.9
2409.1
2597.1
Eumotopias jubatus
1453.7
2975.2
2121.0
1105.5
2853.3
1561.7
1816.8
Otaria flavescens
1453.6
2853.2
1105.5
2120.9
1816.7
2019.8
2342.0
Intens. [a.u.] x10⁴
m/z
1000
1250
1500
1750
2000
2250
2500
2750
3000
Fig S3

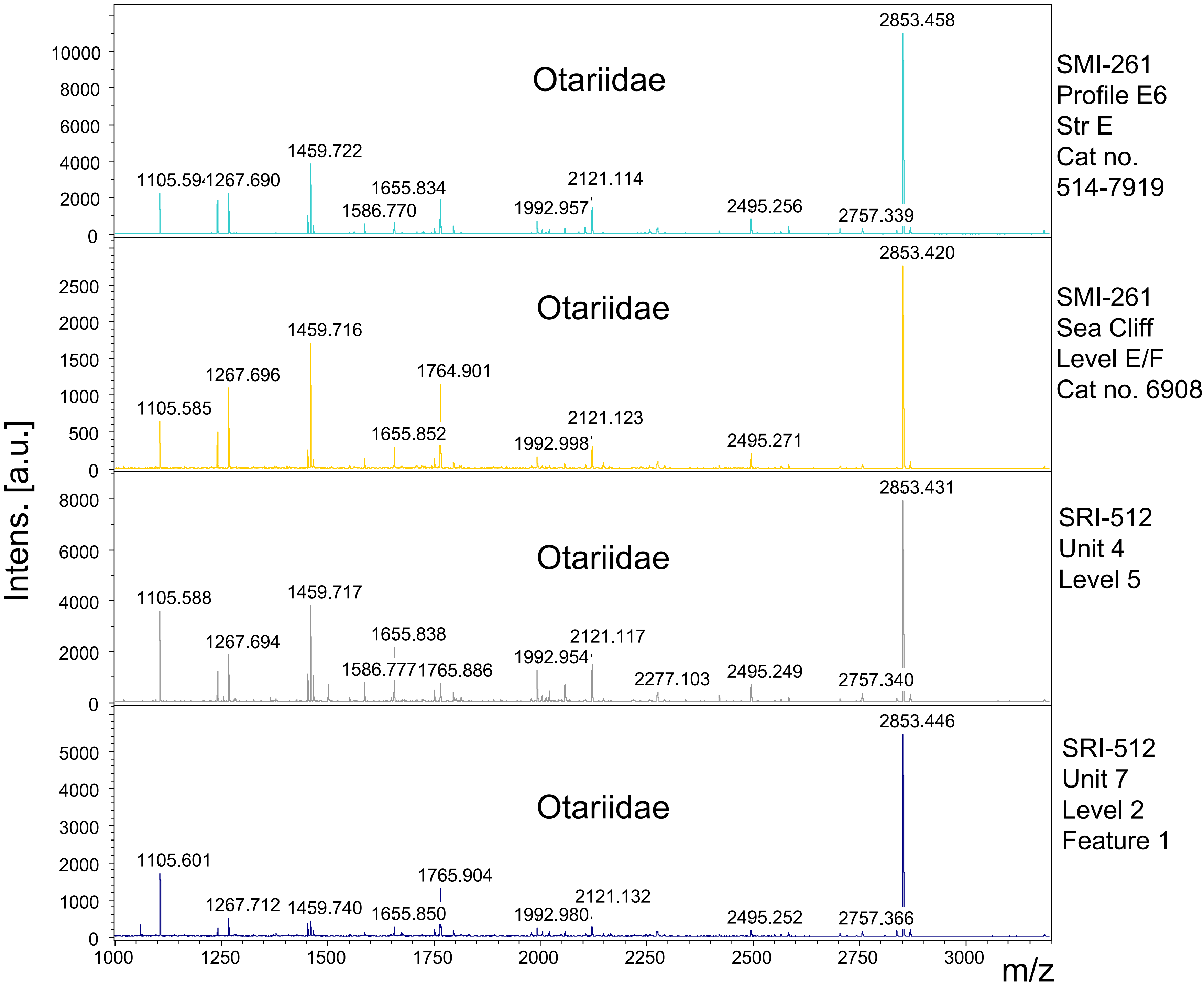
Otariidae
2853.458
1105.594
1267.690
1459.722
1655.834
1586.770
1992.957
2121.114
2495.256
2757.339
SMI-261
Profile E6
Str E
Cat no.
514-7919
Otariidae
2853.420
1105.585
1267.696
1459.716
1764.901
1655.852
1992.998
2121.123
2495.271
SMI-261
Sea Cliff
Level E/F
Cat no. 6908
Otariidae
2853.431
1105.588
1267.694
1459.717
1655.838
1586.777
1765.886
1992.954
2121.117
2277.103
2495.249
2757.340
SRI-512
Unit 4
Level 5
Otariidae
2853.446
1105.601
1267.712
1459.740
1765.904
1655.850
1992.980
2121.132
2495.252
2757.366
SRI-512
Unit 7
Level 2
Feature 1
Intens. [a.u.]
m/z

Fig S4

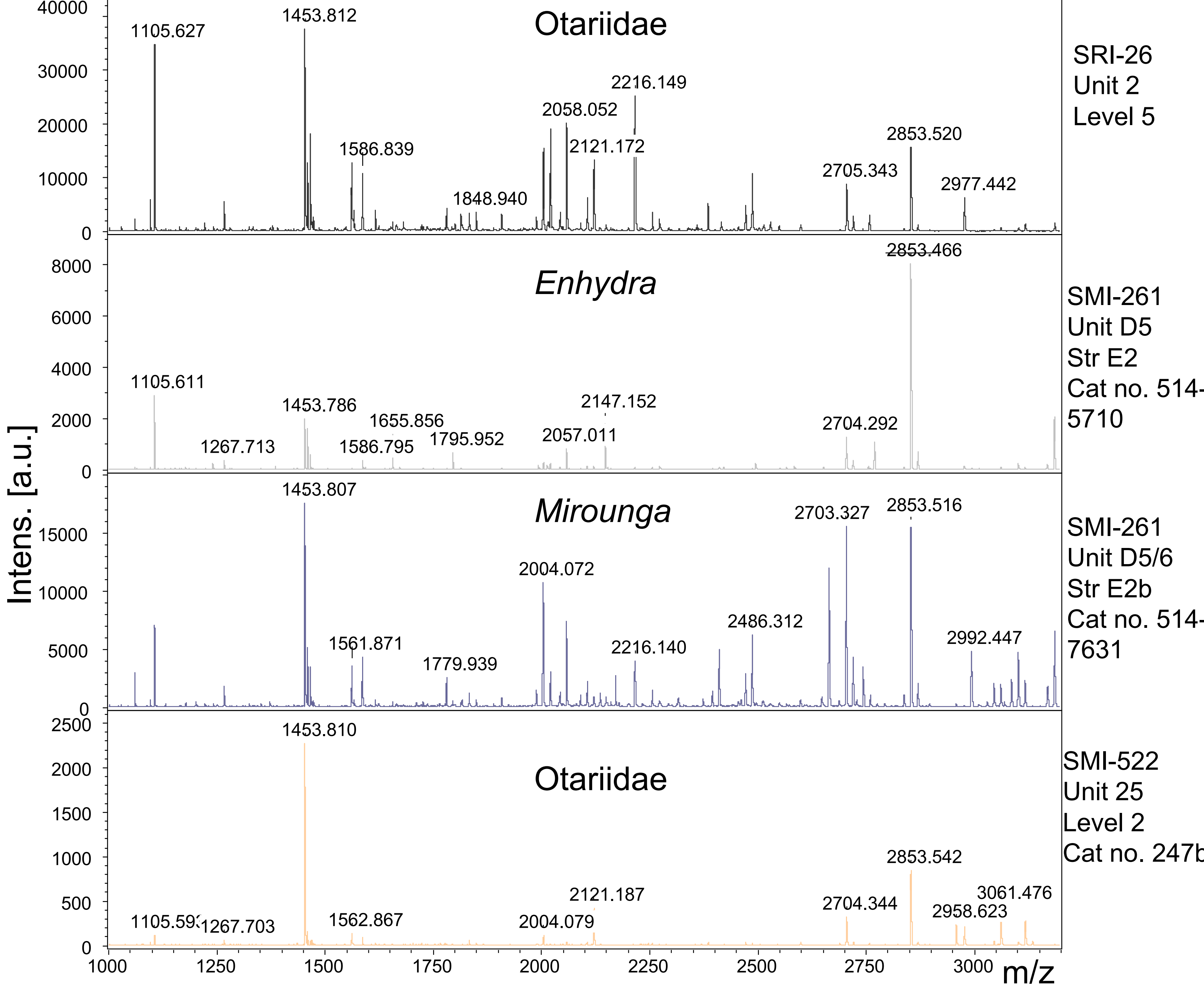
Otariidae
SRI-26
Unit 2
Level 5
1105.627
1453.812
1586.839
1848.940
2058.052
2216.149
2121.172
2705.343
2853.520
2977.442
Enhydra
SMI-261
Unit D5
Str E2
Cat no. 514-
5710
2853.466
1105.611
1267.713
1453.786
1655.856
1586.795
1795.952
2147.152
2057.011
2704.292
Mirounga
SMI-261
Unit D5/6
Str E2b
Cat no. 514-
7631
1453.807
1561.871
1779.939
2004.072
2216.140
2486.312
2703.327
2853.516
2992.447
Otariidae
SMI-522
Unit 25
Level 2
Cat no. 247b
1453.810
1267.703
1562.867
2004.079
2121.187
2704.344
2853.542
2958.623
3061.476
Intens. [a.u.]
m/z

Fig S5

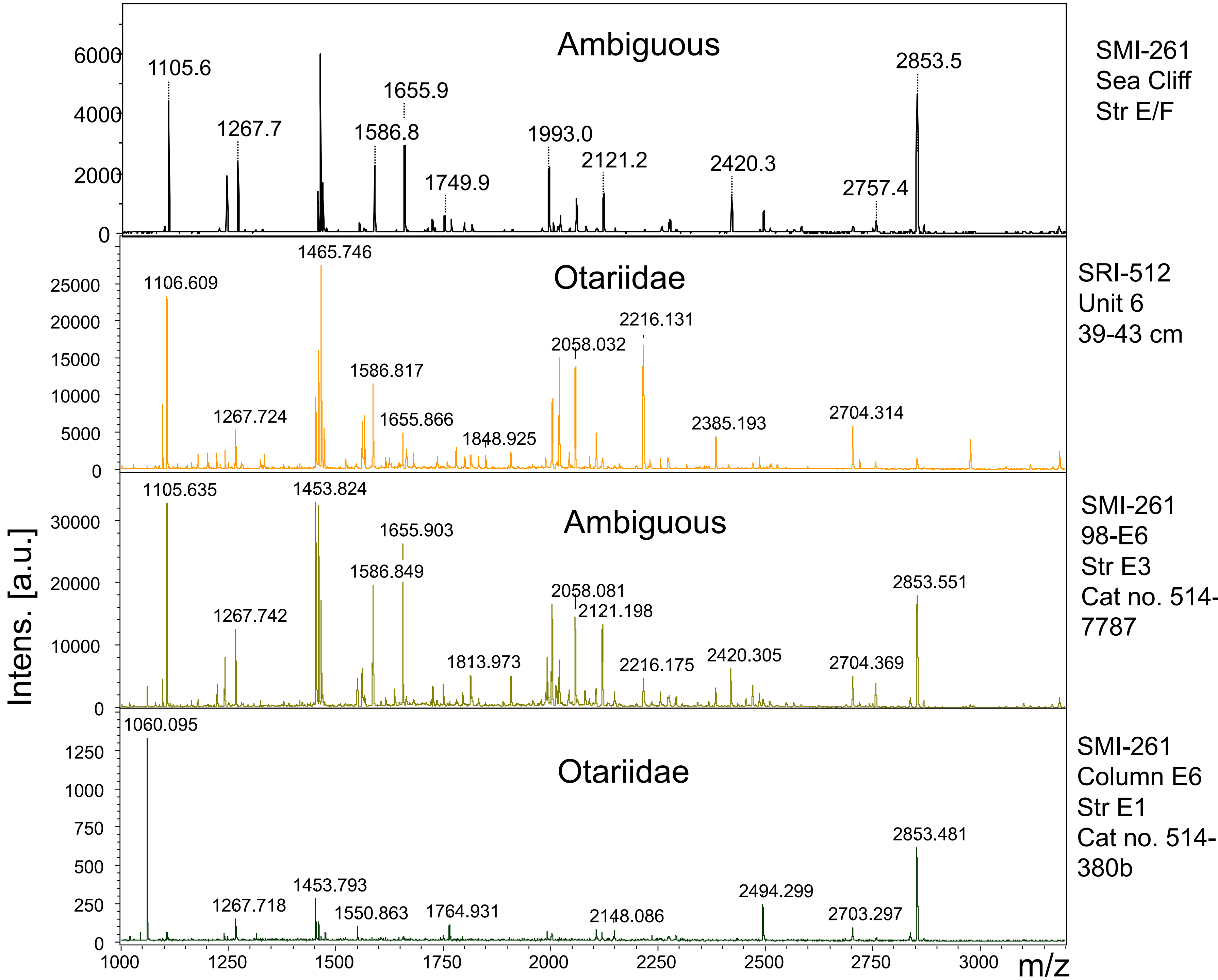
Ambiguous
SMI-261
Sea Cliff
Str E/F
1105.6
1267.7
1655.9
1586.8
1749.9
1993.0
2121.2
2420.3
2757.4
2853.5
Otariidae
SRI-512
Unit 6
39-43 cm
1106.609
1465.746
1267.724
1586.817
1655.866
1848.925
2058.032
2216.131
2385.193
2704.314
Ambiguous
SMI-261
98-E6
Str E3
Cat no. 514-
7787
1105.635
1453.824
1655.903
1586.849
1267.742
1813.973
2058.081
2121.198
2216.175
2420.305
2704.369
2853.551
Otariidae
SMI-261
Column E6
Str E1
Cat no. 514-
380b
1060.095
1267.718
1453.793
1550.863
1764.931
2148.086
2494.299
2703.297
2853.481
Intens. [a.u.]
m/z

Fig S6

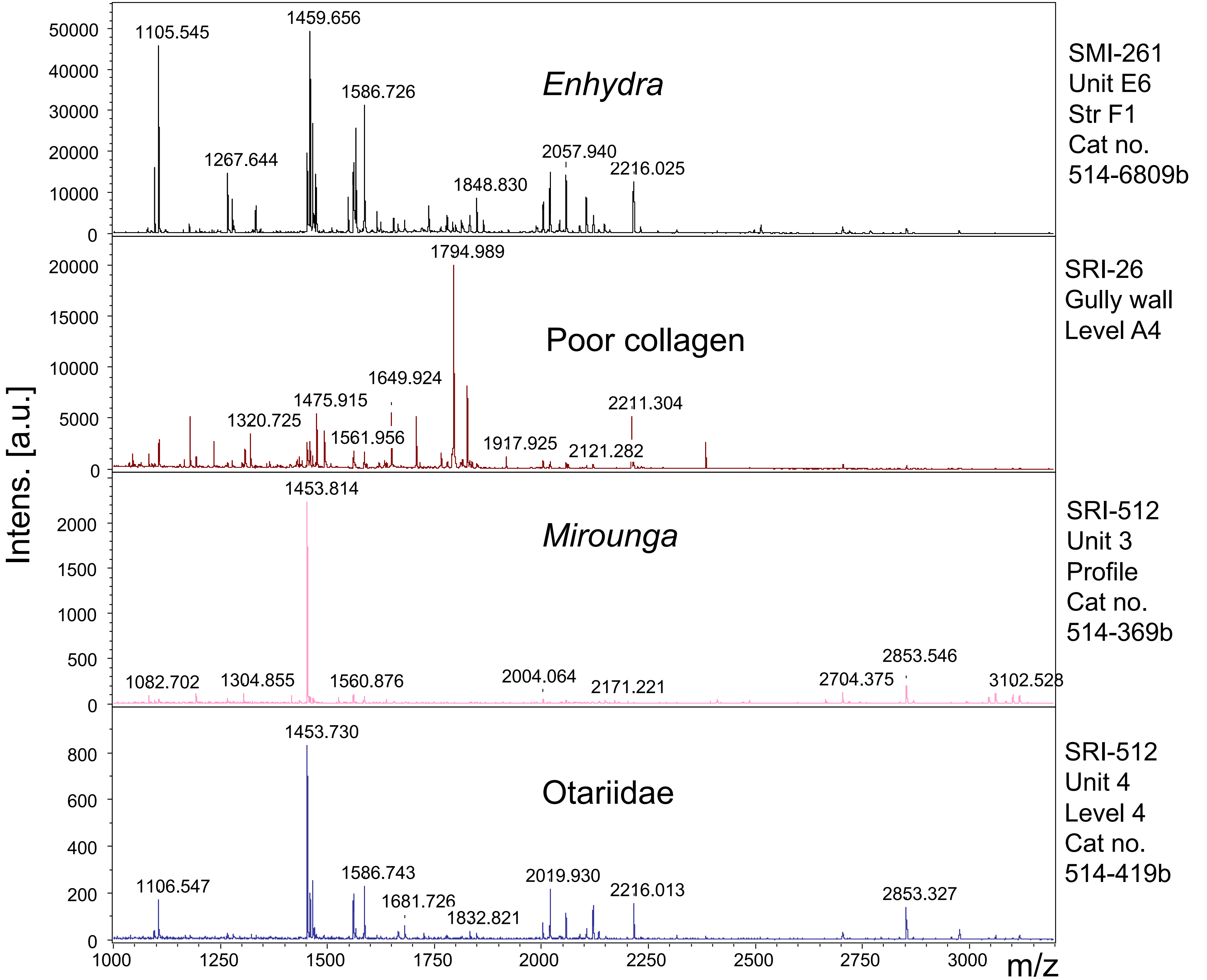

Enhydra
1105.545
1459.656
1586.726
1267.644
1848.830
2057.940
2216.025
SMI-261
Unit E6
Str F1
Cat no.
514-6809b
Poor collagen
1794.989
1649.924
1475.915
1320.725
1561.956
1917.925
2121.282
2211.304
SRI-26
Gully wall
Level A4
Mirounga
1453.814
1082.702
1304.855
1560.876
2004.064
2171.221
2704.375
2853.546
3102.528
SRI-512
Unit 3
Profile
Cat no.
514-369b
Otariidae
1453.730
1106.547
1586.743
1681.726
1832.821
2019.930
2216.013
2853.327
SRI-512
Unit 4
Level 4
Cat no.
514-419b
Intens. [a.u.]
m/z

Fig S7

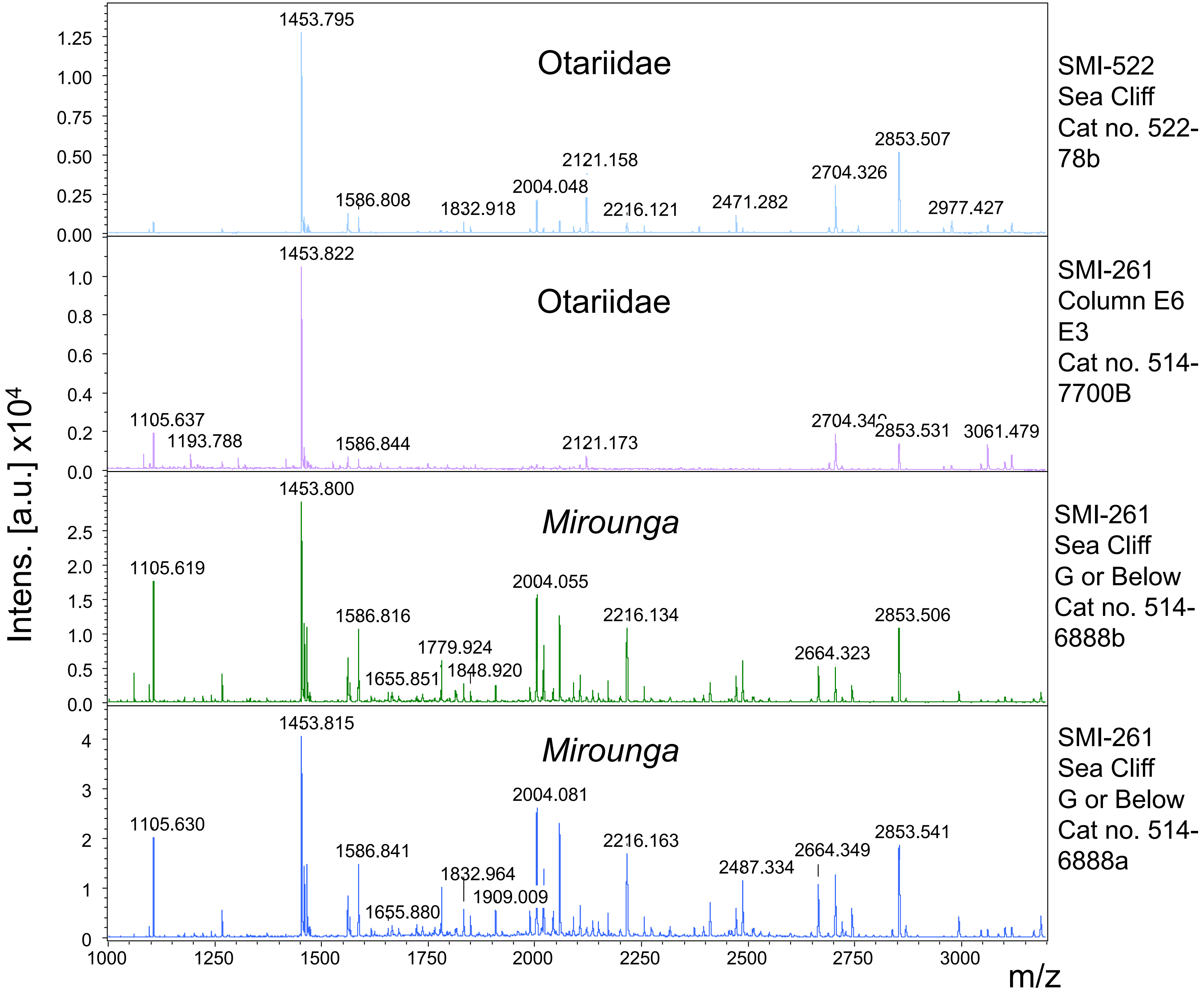
Otariidae
SMI-522
Sea Cliff
Cat no. 522-
78b
1453.795
1586.808
1832.918
2004.048
2121.158
2216.121
2471.282
2704.326
2853.507
2977.427
Otariidae
SMI-261
Column E6
E3
Cat no. 514-
7700B
1453.822
1105.637
1193.788
1586.844
2121.173
2704.34
2853.531
3061.479
Mirounga
SMI-261
Sea Cliff
G or Below
Cat no. 514-
6888b
1453.800
1105.619
1586.816
1655.851
1779.924
1848.920
2004.055
2216.134
2664.323
2853.506
Mirounga
SMI-261
Sea Cliff
G or Below
Cat no. 514-
6888a
1453.815
1105.630
1586.841
1655.880
1832.964
1909.009
2004.081
2216.163
2487.334
2664.349
2853.541
Intens. [a.u.] x10^4
m/z

Fig S8
